# Glycated Albumin is Independently Associated With Arterial Stiffness in Non-Diabetic Chronic Kidney Disease Patients

**DOI:** 10.1097/MD.0000000000003362

**Published:** 2016-04-22

**Authors:** Hoon Young Choi, Seung Kyo Park, Gi Young Yun, Ah Ran Choi, Jung Eun Lee, Sung Kyu Ha, Hyeong Cheon Park

**Affiliations:** From the Department of Internal Medicine, Gangnam Severance Hospital (HYC, SKP, GYY, ARC, SKH, HCP); Severance Institute for Vascular and Metabolic Research, Yonsei University College of Medicine (HYC, HCP), Seoul; and Department of Internal Medicine, Yongin Severance Hospital, Yonsei University College of Medicine (JEL), Gyeongi-do, Korea.

## Abstract

Glycated albumin (GA) exhibits atherogenic effects and increased serum GA levels are associated with the development of cardiovascular complications in diabetic patients. GA production also increases with aging, oxidative stress, and renal dysfunction. We performed this study to further ascertain the association between GA and arterial stiffness in nondiabetic chronic kidney disease (CKD) patients.

We enrolled 129 nondiabetic CKD patients. Arterial stiffness was measured by brachial-ankle pulse wave velocity (baPWV) using a volume plethysmographic instrument along with simultaneous measurements of GA. Insulin resistance was determined with the homeostatic model assessment. The estimated glomerular filtration rate was calculated using serum creatinine and cystatin C according to the CKD-EPI Creatinine-Cystatin C equation adjusted for age, sex, and race (eGFRcr-cys).

Nondiabetic CKD patients with arterial stiffness (baPWV ≥1400 cm/s) showed higher GA levels than those without arterial stiffness (14.2 [8.7–20.2]% vs 13.0 [8.8–18.9]%, *P* *=* 0.004). In the subgroup analysis, the patients who had both a higher GA level and a lower eGFRcr-cys, showed the highest baPWV compared with patients with a higher GA or a lower GFR alone.

By Spearman's correlation analysis, GA correlated significantly with baPWV (*r* = +0.291, *P* = 0.001) and fasting serum glucose level (*r* = +0.191, *P* = 0.030), whereas The homeostatic model assessment of insulin resistance did not show any significant correlation with baPWV. Systolic blood pressure (*r* = +0.401 *P* *<* 0.001), age (*r* = +0.574, *P* *<* 0.001), high-density lipoprotein (HDL)-cholesterol level (*r* = −0.317, *P* *<* 0.001), and eGFRcr-cys (*r* = −0.285, *P* = 0.002) had a significant correlation with baPWV. According to multivariable logistic regression analysis, higher GA and systolic blood pressure were the independent risk factors affecting arterial stiffness.

Our results suggest that serum GA is a potential risk factor of arterial stiffness in nondiabetic CKD patients.

## Abstract 2

Supplemental Digital Content is available in the text

## INTRODUCTION

Cardiovascular disease (CVD) is a major cause of death in chronic kidney disease (CKD) patients.^1,2^ The Second National Health and Nutrition Examination Survey showed that even a mild-to-moderate renal insufficiency was independently associated with subsequent death from CVD.^3,4^ However, this higher cardiovascular morbidity and mortality observed in CKD patients cannot be fully explained by traditional risk factors alone. Inflammation, malnutrition, endothelial dysfunction, and albuminuria are frequently observed in CKD patients and they have been reported as nontraditional risk factors contributing to increased cardiovascular mortality rate.^5^

Increased arterial stiffness plays an important role in the causation of CVD in CKD patients and is regarded as a nontraditional risk factor for CVD in CKD patients. Moreover, several studies have suggested that arterial stiffness itself may also contribute to the progression of CKD.^6–8^ Arterial stiffness in CKD patients is of multifactorial etiologies including old age, vascular calcification, hypertension, inflammation, uremic toxins, and the renin-angiotensin-aldosterone system.^9^ Recent studies have identified accumulation of advanced glycation end products (AGEs) as a novel contributor to arterial stiffening. AGE formation is markedly enhanced in diabetics because of sustained hyperglycemia. In uremia, however, the presence of carbonyl stress contributes to AGE modification of proteins, and plasma AGE levels rise dramatically, independent of hyperglycemia. Therefore, AGE concentrations in excess of normal aging or diabetes are frequently found in uremic plasma, irrespective of the presence of diabetes. The accumulation of AGEs or binding of AGEs to receptors in the vessel wall is associated with the cross-linking of collagen and induction of cell signaling, which result in oxidative stress, increased expression of cytokines and adhesion molecules, and activation of nuclear factor–kappa B (NF-kB). The augmentation of these events would result in the increased arterial stiffness observed in CKD patients.^10–13^

Glycated albumin (GA), a precursor for generation of AGEs, is the major form of circulating Amadori-type glycated proteins. It has been reported to be a better glycemic indicator than glycated hemoglobin (HbA_1_C) in hemodialysis patients with diabetes. In addition to its role as a sensitive marker of glycemic status, there is growing evidence that GA is associated with increased oxidative stress and endothelial injury resulting in diabetic vasculopathy.^14^ Increased GA concentrations are associated with accelerated atherosclerosis as well as presence and severity of coronary artery disease in type 2 diabetic patients.^14–17^ Furthermore, GA demonstrated significant positive correlation with arterial stiffness measured by brachial-ankle pulse wave velocity (baPWV) values in diabetic hemodialysis patients.^18^ These studies support the role of GA in predicting diabetic vascular complications.

In nondiabetic participants with mild-to-advanced CKD, GA levels were shown to have a significant negative correlation with glomerular filtration rate (GFR) and were independent of HbA_1_C, hematocrit, systolic blood pressure, body mass index (BMI), and the amount of proteinuria.^19^ Few clinical studies have examined the role of GA in predicting nondiabetic vascular complications. Therefore, we aimed to investigate whether elevated GA levels are associated with increased arterial stiffness in nondiabetic CKD patients.

## METHODS

### Participants

One hundred twenty-nine nondiabetic CKD patients were enrolled during their routine clinical visits between March 2012 and October 2013. All patients were diagnosed with CKD according to the National Kidney Foundation K/DOQI Guidelines based on an estimated GFR (eGFR) of <60 mL/min/1.73 m^2^ with or without the presence of kidney damage. Kidney damage for >3 months was defined as structural or functional abnormalities of the kidney with or without decreased GFR, manifested by either pathologic abnormalities or markers of kidney damage including abnormalities in the composition of the blood or urine or on imaging tests.^20^ The eGFR was calculated by serum creatinine and cystatin C using the CKD-EPI creatinine-cystatin C equation adjusted for age, sex, and race.^21^ A medical history and a physical examination were performed to gather additional information, including sex, underlying disease that may have caused CKD, comorbidity including cardiovascular disease and cerebrovascular disease, use of antihypertensive agents (such as calcium channel blockers, angiotensin-receptor antagonists, or angiotensin-converting enzyme inhibitors), statin use, blood pressure, and BMI (kg/m^2^). The laboratory findings measured on the day of baPWV assessment such as hemoglobin, fasting serum glucose, insulin, albumin, total cholesterol, triglycerides, high-density lipoprotein (HDL)-cholesterol, low-density lipoprotein (LDL)-cholesterol, serum creatinine, blood urea nitrogen, cystatin C, urine protein-creatinine ratio (UPCR), and GA were included in this study. Serum GA levels were determined using an enzymatic method (Hitachi 7600 P module auto-analyzer, Hitachi Instruments Service, Tokyo, Japan). The coefficient of variation for GA was 2.49% at 0.6 g/dL and 1.81% at 1.5 g/dL. Insulin resistance was calculated by the homeostatic model assessment of insulin resistance (HOMA-IR) index (plasma glucose [mmol] × [plasma insulin level/22.5]).^22,23^

This study was approved by the Institutional Review Board (3–2014–0234) of Gangnam Severance Hospital, Yonsei University College of Medicine, Seoul, Korea.

### Pulse Wave Velocity Measurement

Arterial stiffness was assessed by baPWV, as previously reported.^24^ Patients had abstained from caffeine and any other medications, including antihypertensive agents, for at least 12 hours before the baPWV assessment. The baPWV measurements were obtained at the bedside of each participant using a volume plethysmographic instrument (VP 1000, OMRON Healthcare, Kyoto, Japan). Assessments of both baPWV values were performed after at least 5 minutes of rest in the supine position in an air-conditioned room (24°C ∼26°C). For our analysis, the mean baPWV was used. The validity, reliability, and reproducibility of this instrument were assessed in a previous study.^24^ The participants with baPWV value of ≥1400 cm/s were defined as “Stiffness” group, whereas those with baPWV <1400 cm/s were grouped as “non-stiffness” group, as previously reported.^25^

### Statistical Analysis

Statistical analyses were performed using the Statistical Package for the Social Sciences (SPSS^®^) version 20.0 (SPSS Inc, Chicago, IL). All continuous variables are described as the median (interquartile range) based on results from Kolmogorov-Smirnov tests, which identified data that were not normally distributed. Categorical data are expressed as the number with percentages. Statistical comparisons between groups were performed using Mann-Whitney *U* tests or *χ*^2^ tests, both nonparametric statistical methods. Spearman correlation coefficient was used for comparisons between pairs of variables. The predictive values of HOMA-IR, fasting glucose, and GA for increased baPWV were calculated by constructing receiver-operating characteristic (ROC) curves, and comparison of the ROC curves was performed using the Delong method. The value of the cutoff point for GA for predicting arterial stiffness was calculated using the Youden method.^26^

Multivariable logistic regression analyses were used to estimate multiple correlations between arterial stiffness and clinical and laboratory risk factors. A *P* value of <0.05 was considered to be statistically significant. *P* values were calculated after (Holm)-Bonferroni correction for multiple testing.

## RESULTS

### Characteristics of Participants

The baseline characteristics of participants are shown in Table 1. In all 129 nondiabetic CKD patients (62 men and 67 women) with a median age of 58 (29–82) years were included in this study. The causes of CKD were 60 cases of hypertension (45.7%), 33 cases of glomerulonephritis (25.6%; IgA nephropathy: 23, minimal change disease: 3, ANCA-associated glomerulonephritis: 3, membranous nephropathy: 1, focal segmental glomerulosclerosis: 2, post-streptococcal glomerulonephritis: 1), 7 cases of other conditions such as polycystic kidney disease (5.4%), and 29 cases with an unknown etiology (22.5%). One hundred eleven patients had taken anti-hypertensive medications such as calcium channel blockers (CCB), angiotensin II-receptor blockers (ARB) or angiotensin-converting enzyme inhibitors (ACEi) or some combination of these medications. Eighty-three patients had been prescribed statins. The median level of eGFRcr-cys was 54 mL/min/1.73 m^2^ and the median GA levels were 13.6%. The median baPWV was 1456.5 cm/s. Patient characteristics for males and females are included in sTable 1.

**TABLE 1:**
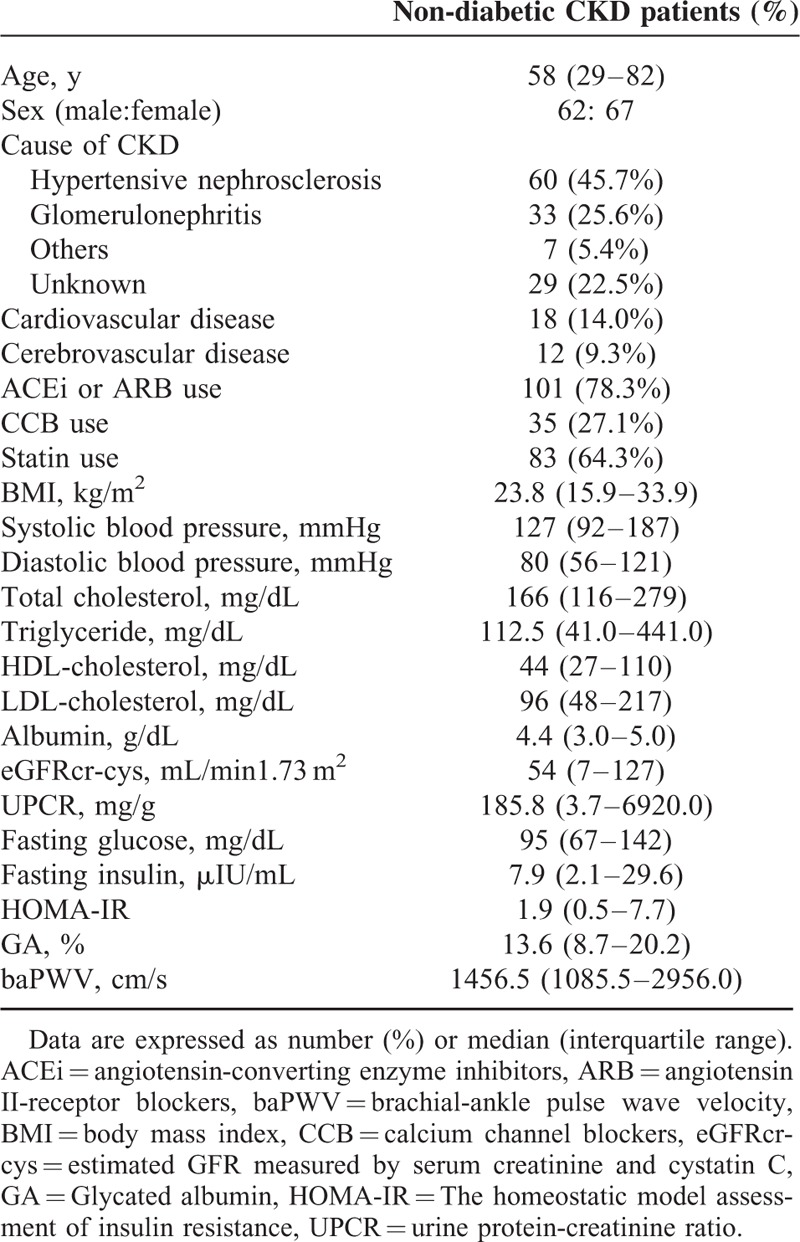
Characteristics of the Whole Study Participants

Table 2 shows the clinical characteristics and biochemical findings of the patients who were classified by arterial stiffness. Seventy-five patients (58.1%) reported increased arterial stiffness (baPWV ≥400 cm/s, “stiffness group”). Age, the number of patients with CVD systolic blood pressure, and baPWV were significantly higher, whereas HDL-cholesterol and eGFRcr-cys were lower in the “stiffness” group than in the “non-stiffness” group. The “stiffness” group showed higher GA levels than the “non-stiffness” group (14.2 [8.7–20.2]% vs 13.0 [8.8–18.9]%, *P* *=* 0.004, Table 2). However, other glycemic indices, including fasting glucose, insulin, and HOMA-IR did not reveal any significant differences between the 2 groups (Table 2).

**TABLE 2:**
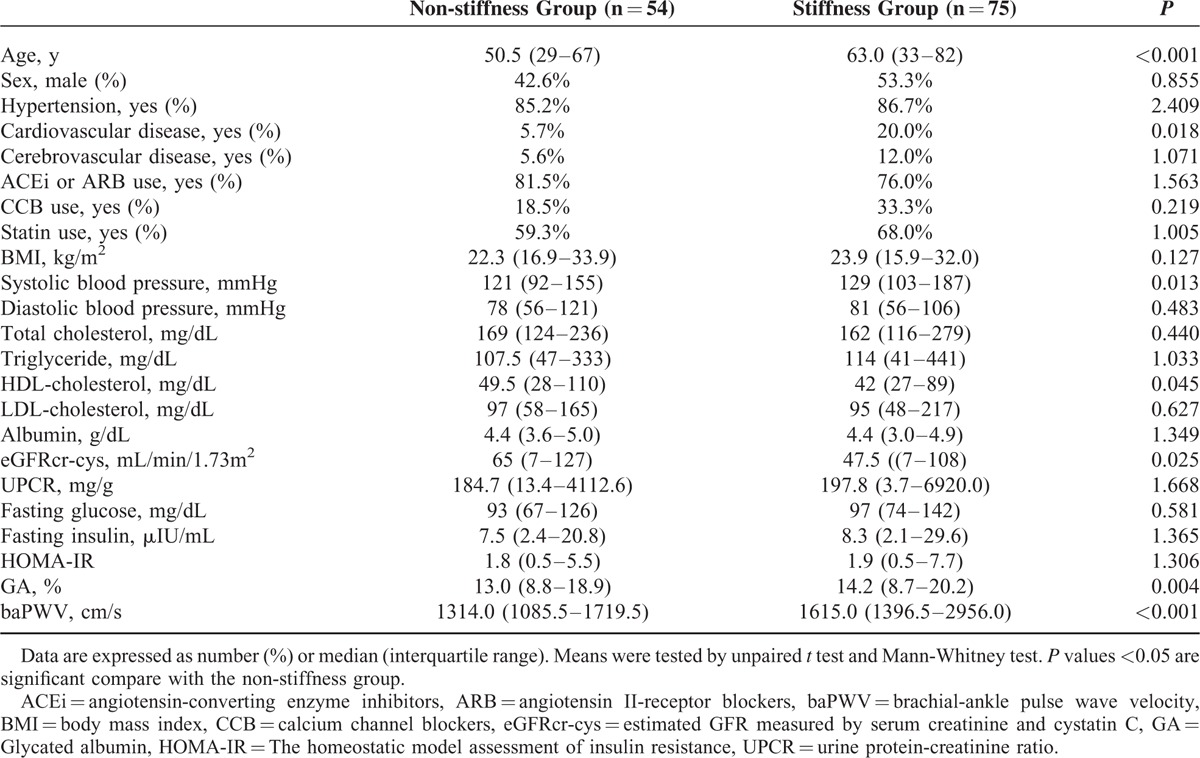
Participant Characteristics Classified by Arterial Stiffness

**FIGURE 1:**
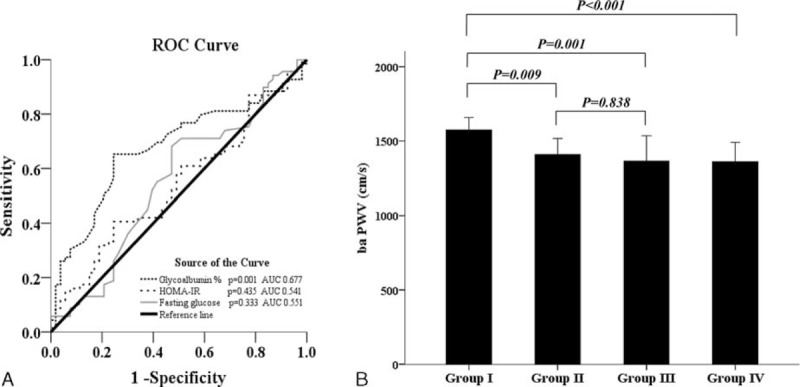
Receiver-operating characteristic (ROC) curve and Brachial–ankle pulse wave velocity in subgroups. ROC curve of each glycemic indices predicting arterial stiffness (A). Brachial–ankle pulse wave velocity in subgroups. Group I: higher glycated albumin (GA) and lower glomerular filtration rate (GFR); Group II: higher GA and higher GFR; Group III: lower GA and lower GFR; Group IV: lower GA and higher GFR (B).

We also constructed receiver-operating characteristics (ROC) curves to predict arterial stiffness based on GA levels or other glycemic indices. The area under the ROC curve (AUC) of GA levels for arterial stiffness was significantly larger than that of HOMA-IR or fasting glucose levels (AUC of GA levels = 0.677; 95% CI, 0.581–0.773 vs AUC of HOMA-IR = 0.541; 95% CI, 0.439–0.644, AUC of fasting glucose levels = 0.551; 95% CI, 0.446–0.656) (Figure 1A). According to the Youden method, the value of the cutoff point for GA was 13.6% for predicting arterial stiffness in all participants (sensitivity [95% CI]: 64 [52.1–74.8]; specificity [95% CI]: 75.9 [62.4–86.5]; PPV [95% CI]: 76.2 [65.7–86.7]; NPV [95% CI]: 59.1 [47.3–71.0]).

### Subgroup Analyses According to GA and Renal Function

We classified all participants according to their GA levels. Table 3 shows the characteristics and biochemical findings of the participants who were grouped by their GA levels. Sixty-four patients (49.6 %) had higher GA levels than the cutoff point of GA (≥13.6%). Age was significantly higher and eGFRcr-cys were lower in the “Higher GA” group than in the “Lower GA” group. The “Higher GA” group showed significant arterial stiffness compared with that of the “Lower GA” (baPWV 1534.8 [1096.0–2956.0] vs 1360.5 [1085.5–2219.5] cm/s, *P* *<* 0.001) (Table 3).

**TABLE 3:**
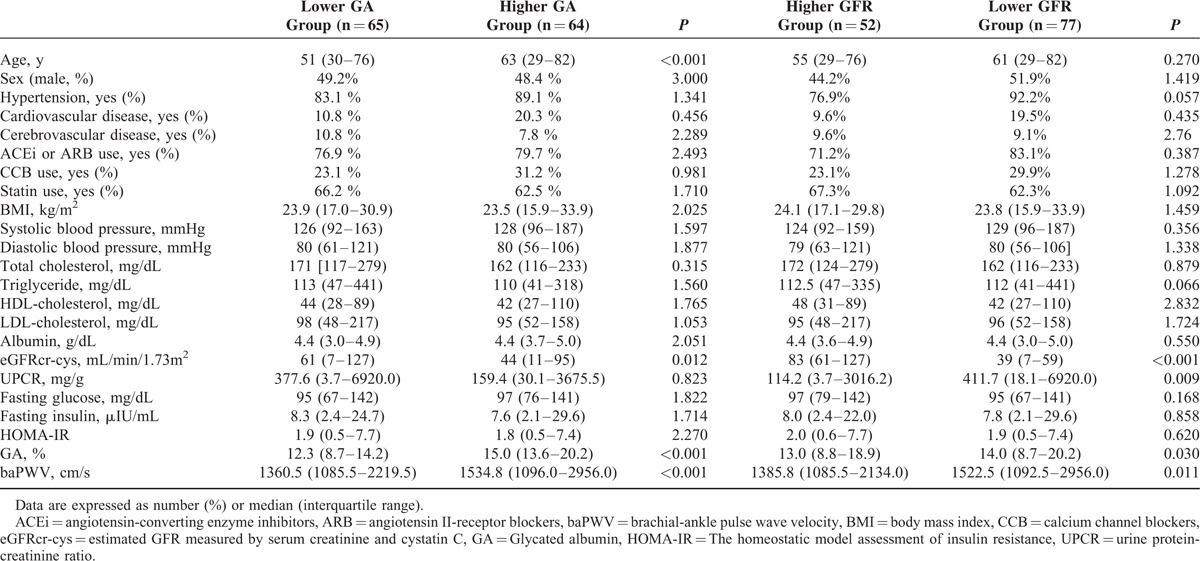
Participant Characteristics Classified by GA Levels or by GFR

Next, 52 patients (40.3%) were classified in the “Lower GFR group” (eGFRcr-cys less than 60 mL/min/1.73 m^2^), whereas 77 patients (59.7%) were classified in the “Higher GFR group” (eGFRcr-cys greater than 60 mL/min/1.73 m^2^). In the “Lower GFR” group, UPCR and GA levels were higher, and eGFRcr-cys values were lower than those in the “Higher GFR” group (Table 3). Similar to the patients with higher GA levels, the “Lower GFR” group also revealed a significantly higher baPWV than the “Higher GFR” group (baPWV 1522.5 [1092.5–2956.0] vs 1385.8 [1085.5–2134.0] cm/s, *P* = 0.011) (Table 3).

To investigate the compound effects of renal function and higher GA levels of patients on arterial stiffness, the patients were classified into 4 groups (Group I: participants with a GA >13.6% and eGFRcr-cys <60 mL/min/1.73 m^2^; Group II: participants having a GA >13.6% and eGFRcr-cys ≥60 mL/min/1.73 m^2^; Group III: participants with a GA ≤13.6% and eGFRcr-cys <60 mL/min/1.73 m^2^; Group IV: participants having a GA ≤13.6% and eGFRcr-cys ≥60 mL/min/1.73 m^2^). As a result, the patients who had higher GA levels with a lower eGFR (Group I) showed the highest baPWV among the 4 subgroups (Group I: 1573.5 [1206.0–2956.0] cm/s; Group II: 1408.8 [1096.0–2034.0] cm/s; Group III: 1364.3 [1092.5–2219.5] cm/s; Group IV: 1360.0 [1085.5–2134.0] cm/s, *P* < 0.001). However, no significant differences were found between the group with a lower GA and a higher GFR and the group with a higher GA alone or the group that only had lower eGFR (*P* = 0.838) (Figure 1B).

### Association of baPWV with Glycemic Indices and Cardiovascular Risk Factors

**FIGURE 2:**
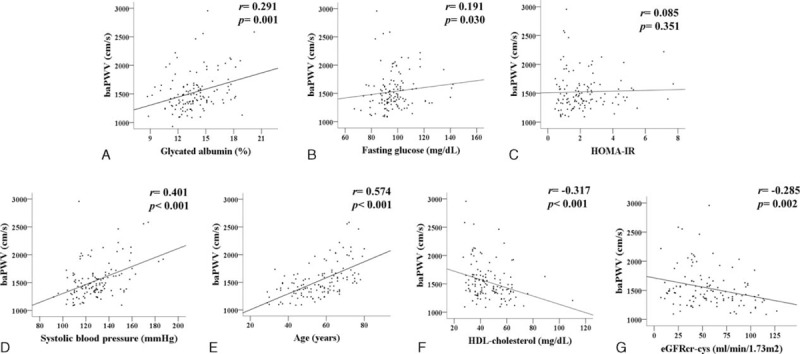
Correlation between brachial–ankle pulse wave velocity and glycated albumin (A), fasting glucose (B), HOMA-IR (C), systolic blood pressure (D), age (E), HDL-cholesterol (F), eGFRcr-cys (G) in the entire subjects. baPWV; brachial–ankle pulse wave velocity, HOMA-IR; The homeostatic model assessment of insulin resistance, HDL: high-density lipoprotein, eGFRcr-cys; estimated GFR measured by serum creatinine and cystatin C. baPWV = brachial-ankle pulse wave velocity, eGFRcr-cys = estimated glomerular filtration rate measured by serum creatinine and cystatin C, HDL = high-density lipoprotein, HOMA-IR = homeostatic model assessment of insulin resistance.

Figure 2 shows the Spearman analysis coefficients between baPWV and the variables for glycemic indices and cardiovascular risk in the enrolled participants. In all participants, baPWV correlated significantly with GA (*r* = +0.291, *P* = 0.001) and fasting glucose level (*r* = +0.191, *P* = 0.030), whereas HOMA-IR did not show any significant correlation with baPWV (Figure 2A, B, and C). Systolic blood pressure (*r* = +0.401 *P* *<* 0.001) and age (*r* = +0.574, *P* *<* 0.001) were significantly associated with baPWV (Figure 2D, E). HDL-cholesterol level (*r* = −0.317, *P* *<* 0.001) and eGFRcr-cys (*r* = −0.285, *P* = 0.002) showed a significant negative correlation with baPWV (Figure 2F, G).

For multivariable logistic regression analysis, higher GA, lower eGFRcr-cys, fasting glucose, and other conventional risk factors, including age group with cardiovascular risk (male >45 years, female >55 years), male sex, systolic blood pressure, and serum HDL-cholesterol, were entered as the independent variables affecting arterial stiffness. Higher GA (OR 2.883, *P* = 0.015) and systolic blood pressure (OR 1.034, *P* = 0.015) were significant independent factors affecting arterial stiffness (Table 4).

**TABLE 4:**
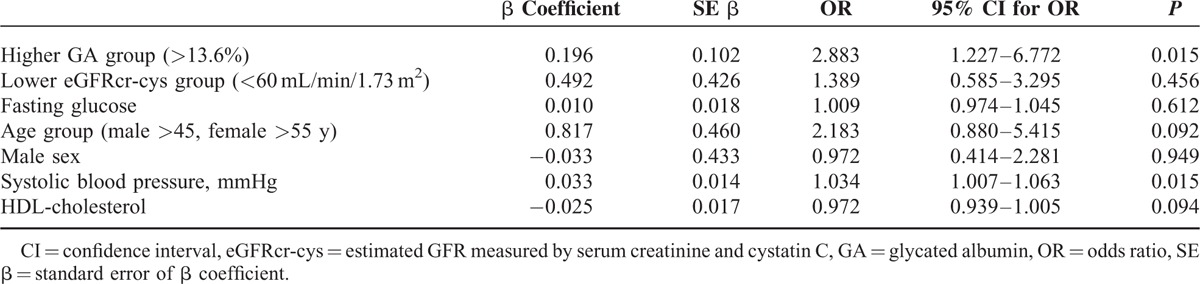
Logistic Regression Analysis for the Determinants of Arterial Stiffness

## DISCUSSION

CKD patients face exceptionally increased CVD morbidity and mortality that is characterized by arteriosclerosis and increased arterial stiffness.^8^ This increased arterial stiffness in CKD patients cannot be fully explained by conventional risk factors alone. Nontraditional risk factors, such as inflammation, endothelial dysfunction, and bone mineral derangements, have been implicated in the development of arterial stiffness in CKD.^27^ Furthermore, recent efforts have focused on identifying additional factors that may account for the link between CKD and arterial stiffness.

The present study demonstrated that GA was an independent risk factor for increased arterial stiffness in nondiabetic CKD patients and those with an increased GA and impaired renal function had higher arterial stiffness than those who only demonstrated increased GA or impaired renal function alone.

The formation of AGEs is an important pathway in the development of arterial stiffness in diabetic patients. Recent experimental studies have proposed that GA may also play a role in the mechanism of increased arterial stiffness in diabetes. In vitro experiments have demonstrated that GA promotes inflammation via NF-kB activation in endothelial cells and stimulates vascular smooth muscle cell proliferation.^14,28,29^ Moreover, increased GA has been found to upregulate NADPH oxidase and induce sustained reactive oxygen species production in human endothelial cells.^30^ Diabetic db/db mice treated with anti-GA antibodies normalized plasma levels of fibronectin, a marker of endothelial damage, despite persistent hyperglycemia.^31^ A recent cross-sectional study demonstrated that GA was associated with carotid intima-media thickness (IMT) in patients with type 2 diabetes mellitus without any coronary heart disease and peripheral artery disease.^32^ Moreover, a retrospective longitudinal study has shown that the serum level of GA predicted progression of carotid IMT in patients with type 2 diabetes mellitus after adjustment of common atherogenic risk factors such as age, sex, and LDL-cholesterol.^15^ Taken together, GA could be considered to be an atherogenic protein in the development of diabetic atherosclerosis.^15,32^ Although there is no direct histologic evidence demonstrating GA involvement in arterial stiffness, the above findings suggest that increased GA causally contributes to diabetic vasculopathy. The present study identified a relationship between GA and PWV independent of fasting glucose and insulin resistance in non-diabetic CKD patients. These results suggest that GA could be a biomarker of arterial stiffness beyond glucose metabolism in nondiabetic CKD patients.

Ma et al implied that the impact of GA to eGFR could be masked because the glycemic status strongly affects the GA concentration in diabetic patients. They demonstrated that GA concentrations were significantly correlated with GFR only in nodiabetic CKD patients even after adjusting for proteinuria, hematocrit, HbA_1_C, systolic blood pressure, BMI, and total cholesterol in multivariate analysis.^15,20^ Our results showed that participants with higher GA levels had significantly lower eGFRcr-cys and also demonstrated the significant negative association between eGFRcr-cys and GA levels in nondiabetic CKD patients (data not shown). However, we did not find any significant association between eGFRcr-cys and baPWV after adjusting GA levels, age, sex, and HDL-cholesterol levels. GA might be considered as a powerful risk factor affecting on arterial stiffness in non-diabetic CKD patients regardless of renal function in the present study.

GA was also reported to stimulate TGF-β and increase oxidative stress, which could induce vasculopathy in experimental models.^29^ Therefore, the accumulation of GA as a result of impaired renal clearance was assumed to play an important role in the progression of vasculopathy in CKD patients via the promotion of oxidative stress, production of inflammatory cytokines, and endothelial damage.^14^

On the contrary, several recent studies have reported that the association of arterial stiffness with different stages of CKD or GFR was unsubstantiated. Although the univariate analysis showed a significant association of arterial stiffness and GFR, traditional risk factors for CVD might be more important determinants of arterial stiffness than GFR in multivariate analysis.^25,36,37^ In stage 3 CKD patients, eGFR was not an independent determinant, but traditional risk factors including age, mean arterial pressure, and diabetes were the strongest independent determinants of a higher PWV.^33^ Sengstock et al reported a significant relationship between GFR and PWV, but only 2% of the variation in PWV could be explained by GFR in multivariate analysis when conventional determinants of arterial stiffness, including age, systolic blood pressure, diabetes, and obesity, were analyzed as covariates.^34–36^ These results suggested that previous reports regarding the relationship of renal insufficiency and arterial stiffness might be attributable to common underlying CVD risk factors in CKD patients. The present study also demonstrated that GFR could not be an independent determinant of arterial stiffness in nondiabetic CKD patients in multivariable regression analysis.

To investigate which factors profoundly influence increased arterial stiffness in nondiabetic CKD patients, we performed a subgroup analysis of the PWV according to the GA levels and eGFR. The subgroup with both an increased GA and impaired renal function also had the highest PWV compared with the other subgroups. Interestingly, GA levels showed a significantly positive correlation only in CKD patients with impaired renal function compared with those in CKD patients with eGFRcr-cys ≥60 mL/min/1.73 m^2^. Taken together, these findings suggested that nondiabetic CKD patients with increased GA and impaired renal function should be closely monitored for the development of arterial stiffness.

There are several limitations to this report. Our observations were cross-sectional in nature with a small sample size. Therefore, a lack of understanding regarding the exact relationship between GA and arterial stiffness still exists. Prospective studies should be performed to determine whether increased GA can predict the progression of arterial stiffness in CKD. Second, urinary glycosylated protein levels were not obtained; therefore, we could not determine whether increased GA levels were induced by impaired renal clearance or glycemic status. However, only nondiabetic CKD patients with lower mean GA levels than those reported for diabetic patients were enrolled in this study. Therefore, increased GA itself could be meaningful in the progression of vasculopathy as a risk for CVD in nondiabetic CKD patients, regardless of impaired renal clearance. Finally, baPWV was used as a measurement of arterial stiffness instead of carotid-femoral PWV (cfPWV).^37,38^ Recently, baPWV has been shown to represent both central and peripheral arterial stiffness and is regarded as a useful tool for evaluating the risk assessment of CVD towing to the ease of the measurement technique relative to that of cfPWV.^38^

In conclusion, GA showed a significant correlation with baPWV and was the independent risk factor affecting baPWV in nondiabetic CKD patients. Additionally, patients who have higher GA levels together with lower eGFRcr-cys should be monitored for arterial stiffness carefully. The measurement of serum GA concentrations may be predictive of arterial stiffness in nondiabetic CKD patients.

## Supplementary Material

Supplemental Digital Content

## References

[1] Foley RN Parfrey PS Sarnak MJ Clinical epidemiology of cardiovascular disease in chronic renal disease. *Am J Kidney Dis* 1998; 32 (5 Suppl 3):S112–S119. 9820470 10.1053/ajkd.1998.v32.pm9820470

[2] Shinohara K Shoji T Tsujimoto Y Arterial stiffness in predialysis patients with uremia. *Kidney Int* 2004; 65:936–943. 14871413 10.1111/j.1523-1755.2004.00468.x

[3] Muntner P He J Hamm L Renal insufficiency and subsequent death resulting from cardiovascular disease in the United States. *J Am Soc Nephrol* 2002; 13:745–753. 11856780 10.1681/ASN.V133745

[4] Wang MC Tsai WC Chen JY Stepwise increase in arterial stiffness corresponding with the stages of chronic kidney disease. *Am J Kidney Dis* 2005; 45:494–501. 15754271 10.1053/j.ajkd.2004.11.011

[5] Zoccali C Traditional and emerging cardiovascular and renal risk factors: an epidemiologic perspective. *Kidney Int* 2006; 70:26–33. 16723985 10.1038/sj.ki.5000417

[6] Taal MW Sigrist MK Fakis A Markers of arterial stiffness are risk factors for progression to end-stage renal disease among patients with chronic kidney disease stages 4 and 5. *Nephron Clin Pract* 2007; 107:c177–c181. 17975325 10.1159/000110678

[7] Ford ML Tomlinson LA Chapman TP Aortic stiffness is independently associated with rate of renal function decline in chronic kidney disease stages 3 and 4. *Hypertension* 2010; 55:1110–1115. 20212269 10.1161/HYPERTENSIONAHA.109.143024

[8] Taal MW Arterial stiffness in chronic kidney disease: an update. *Curr Opin Nephrol Hypertens* 2014; 23:169–173. 24389732 10.1097/01.mnh.0000441153.40072.e0

[9] Nemcsik J Kiss I Tisler A Arterial stiffness, vascular calcification and bone metabolism in chronic kidney disease. *World J Nephrol* 2012; 1:25–34. 24175239 10.5527/wjn.v1.i1.25 PMC3782208

[10] Jandeleit-Dahm K Cooper ME The role of AGEs in cardiovascular disease. *Curr Pharm Des* 2008; 14:979–986. 18473849 10.2174/138161208784139684

[11] Choi KM Yoo HJ Kim HY Association between endogenous secretory RAGE, inflammatory markers and arterial stiffness. *Int J Cardiol* 2009; 132:96–101. 18190981 10.1016/j.ijcard.2007.10.047

[12] Semba RD Najjar SS Sun K Serum carboxymethyl-lysine, an advanced glycation end product, is associated with increased aortic pulse wave velocity in adults. *Am J Hypertens* 2009; 22:74–79. 19023277 10.1038/ajh.2008.320 PMC2637811

[13] Miyata T van Ypersele de Strihou C Kurokawa K Alterations in nonenzymatic biochemistry in uremia: origin and significance of “carbonyl stress” in long-term uremic complications. *Kidney Int* 1999; 55:389–399. 9987064 10.1046/j.1523-1755.1999.00302.x

[14] Cohen MP Ziyadeh FN Chen S Amadori-modified glycated serum proteins and accelerated atherosclerosis in diabetes: pathogenic and therapeutic implications. *J Lab Clin Med* 2006; 147:211–219. 16697768 10.1016/j.lab.2005.12.006 PMC1800931

[15] Song SO Kim KJ Lee BW Serum glycated albumin predicts the progression of carotid arterial atherosclerosis. *Atherosclerosis* 2012; 225:450–455. 23040867 10.1016/j.atherosclerosis.2012.09.005

[16] Furusyo N Koga T Ai M Plasma glycated albumin level and atherosclerosis: results from the Kyushu and Okinawa Population Study (KOPS). *Int J Cardiol* 2013; 167:2066–2072. 22658569 10.1016/j.ijcard.2012.05.045

[17] Shen Y Pu LJ Lu L Glycated albumin is superior to hemoglobin A1c for evaluating the presence and severity of coronary artery disease in type 2 diabetic patients. *Cardiology* 2012; 123:84–90. 23018602 10.1159/000342055

[18] Kumeda Y Inaba M Shoji S Significant correlation of glycated albumin, but not glycated haemoglobin, with arterial stiffening in haemodialysis patients with type 2 diabetes. *Clin Endocrinol (Oxf)* 2008; 69:556–561. 18248645 10.1111/j.1365-2265.2008.03202.x

[19] Ma WY Wu CC Pei D Glycated albumin is independently associated with estimated glomerular filtration rate in nondiabetic patients with chronic kidney disease. *Clin Chim Acta* 2011; 412:583–586. 21172335 10.1016/j.cca.2010.12.013

[20] Part 4. Definition and Classification of Stagesof Chronic Kidney Disease. *American Journal of Kidney Diseases* 2002; 39:S46–S75.

[21] Inker LA Schmid CH Tighiouart H Estimating glomerular filtration rate from serum creatinine and cystatin C. *N Engl J Med* 2012; 367:20–29. 22762315 10.1056/NEJMoa1114248 PMC4398023

[22] Dogra GK Herrmann S Irish AB Insulin resistance, dyslipidaemia, inflammation and endothelial function in nephrotic syndrome. *Nephrol Dial Transplant* 2002; 17:2220–2225. 12454236 10.1093/ndt/17.12.2220

[23] Giannattasio C Mangoni AA Failla M Combined effects of hypertension and hypercholesterolemia on radial artery function. *Hypertension* 1997; 29:583–586. 9040442 10.1161/01.hyp.29.2.583

[24] Shin JY Lee HR Shim JY Significance of high-normal serum uric acid level as a risk factor for arterial stiffness in healthy Korean men. *Vasc Med* 2012; 17:37–43. 22363017 10.1177/1358863X11434197

[25] Yamashina A Tomiyama H Arai T Brachial-ankle pulse wave velocity as a marker of atherosclerotic vascular damage and cardiovascular risk. *Hypertens Res* 2003; 26:615–622. 14567500 10.1291/hypres.26.615

[26] Youden WJ Index for rating diagnostic tests. *Cancer* 1950; 3:32–35. 15405679 10.1002/1097-0142(1950)3:1<32::aid-cncr2820030106>3.0.co;2-3

[27] Guerin AP Pannier B Metivier F Assessment and significance of arterial stiffness in patients with chronic kidney disease. *Curr Opin Nephrol Hypertens* 2008; 17:635–641. 19031658 10.1097/mnh.0b013e32830dcd5c

[28] Huebschmann AG Regensteiner JG Vlassara H Diabetes and advanced glycoxidation end products. *Diabetes Care* 2006; 29:1420–1432. 16732039 10.2337/dc05-2096

[29] Cohen MP Shea E Chen S Glycated albumin increases oxidative stress, activates NF-kappa B and extracellular signal-regulated kinase (ERK), and stimulates ERK-dependent transforming growth factor-beta 1 production in macrophage RAW cells. *J Lab Clin Med* 2003; 141:242–249. 12677169 10.1067/mlc.2003.27

[30] Rodino-Janeiro BK Gonzalez-Peteiro M Ucieda-Somoza R Glycated albumin, a precursor of advanced glycation end-products, up-regulates NADPH oxidase and enhances oxidative stress in human endothelial cells: molecular correlate of diabetic vasculopathy. *Diabetes Metab Res Rev* 2010; 26:550–558. 20818804 10.1002/dmrr.1117

[31] Cohen MP Clements RS Cohen JA Glycated albumin promotes a generalized vasculopathy in the db/db mouse. *Biochem Biophys Res Commun* 1996; 218:72–75. 8573179 10.1006/bbrc.1996.0014

[32] Moon JH Chae MK Kim KJ Decreased endothelial progenitor cells and increased serum glycated albumin are independently correlated with plaque-forming carotid artery atherosclerosis in type 2 diabetes patients without documented ischemic disease. *Circ J* 2012; 76:2273–2279. 22664650 10.1253/circj.cj-11-1499

[33] McIntyre NJ Fluck RJ McIntyre CW Determinants of arterial stiffness in chronic kidney disease stage 3. *PLoS One* 2013; 8:e55444. 23383192 10.1371/journal.pone.0055444 PMC3559556

[34] Sengstock D Sands RL Gillespie BW Dominance of traditional cardiovascular risk factors over renal function in predicting arterial stiffness in subjects with chronic kidney disease. *Nephrol Dial Transplant* 2010; 25:853–861. 19854848 10.1093/ndt/gfp559

[35] Sengstock DM Vaitkevicius PV Supiano MA Arterial stiffness is related to insulin resistance in nondiabetic hypertensive older adults. *J Clin Endocrinol Metab* 2005; 90:2823–2827. 15728211 10.1210/jc.2004-1686

[36] Mackey RH Sutton-Tyrrell K Vaitkevicius PV Correlates of aortic stiffness in elderly individuals: a subgroup of the Cardiovascular Health Study. *Am J Hypertens* 2002; 15 (1 Pt 1):16–23. 11824854 10.1016/s0895-7061(01)02228-2

[37] Laurent S Cockcroft J Van Bortel L Expert consensus document on arterial stiffness: methodological issues and clinical applications. *Eur Heart J* 2006; 27:2588–2605. 17000623 10.1093/eurheartj/ehl254

[38] Meyer ML Tanaka H Palta P Repeatability of Central and Peripheral Pulse Wave Velocity Measures: The Atherosclerosis Risk in Communities (ARIC) Study. *Am J Hypertens* 2016; 29:470–475. 26232036 10.1093/ajh/hpv127 PMC4850900

