# Supplementary material for: Glycated Albumin is Independently Associated With Arterial Stiffness in Non-Diabetic Chronic Kidney Disease Patients
Source: Medicine (Baltimore). 2016 Apr 22;95(16):e3362. doi: 10.1097/MD.0000000000003362 (PMC4845823; doi:10.1097/MD.0000000000003362)
Supplement: Supplemental Digital Content [file medi-95-e3362-s001.pdf]

sTable 1. Characteristics of the whole study participants in each sex

	**Non-diabetic CKD patients-Male**	**Non-diabetic CKD patients-Female**
Age, y	59 [29-82]	57 [29-79]
Cause of CKD		
Hypertensive nephrosclerosis	35 (56.5%)	24 (36.4%)
Glomerulonephritis	14 (22.6%)	19 (28.8%)
Others	2 (3.2%)	5 (7.6%)
Unknown	11 (17.7%)	18 (27.3%)
Cardiovascular disease	14 (22.2%)	6 (9.1%)
Cerebrovascular disease	5 (7.9%)	7 (10.6%)
ACEi or ARB use	54 (85.7%)	47 (71.2%)
CCB use	19 (30.2%)	16 (24.2%)
Statin use	39 (61.9%)	44 (66.7%)
BMI, kg/m^2	24.1 [15.9-29.8]	22.6 [16.9-33.9]
Systolic blood pressure, mmHg	128 [104-187]	126 [92-183]
Diastolic blood pressure, mmHg	82 [62-106]	77 [56-121]
Total cholesterol, mg/dL	167 [116-279]	164 [116-236]
Triglyceride, mg/dL	124 [47-441]	90.0 [41-335]
HDL-cholesterol, mg/dL	40 [28-76]	50 [27-110]
LDL-cholesterol, mg/dL	98 [57-217]	95 [48-165]
Albumin, g/dL	4.5 [3.6-4.9]	4.4 [3.0-5.0]
eGFRcr-cys, ml/min1.73m^2	54 [15-114]	55 [7-127]
UPCR, mg/g	278.7 [18.1-6920.0]	158.6 [3.7-4112.6]
Fasting glucose, mg/dL	98 [72-141]	94 [67-142]
Fasting insulin, mcIU/mL	8.1 [2.1-29.6]	6.9 [2.7-24.7]
HOMA-IR	1.9 [0.5-7.4]	1.7 [0.5-7.7]
GA, %	13.5 [8.7-20.2]	13.6 [8.8-18.2]
baPWV, cm/sec	1486.0 [1092.5-2956.0]	1424.0 [1085.5-2554.0]

Data are expressed as number (%) or median [interquartile range]. ACEi; angiotensin-converting enzyme inhibitors, ARB; angiotensin II-receptor blockers, CCB; calcium channel blockers, BMI; body mass index, eGFRcr-cys; estimated GFR measured by serum creatinine and cystatin C, UPCR; urine protein-creatinine ratio, GA; Glycated albumin, HOMA-IR; The homeostatic model assessment of insulin resistance, baPWV; brachial-ankle pulse wave velocity
